# The feasibility of fermented litter as a feed ingredient for ruminant livestock

**DOI:** 10.5455/javar.2021.h517

**Published:** 2021-06-25

**Authors:** Cahya Setya Utama, Marry Christiyanto

**Affiliations:** Faculty of Animal and Agricultural Sciences, Universitas Diponegoro, Semarang, Indonesia

**Keywords:** Fermentation, litter, fiber, heavy metals, organoleptic, worms

## Abstract

**Objective:**

The feasibility of fermented litter as an alternative feed material for ruminant livestock is measured by organoleptic quality, fiber profile, heavy metal contamination, and the presence of worm eggs. This study aimed to examine the influence of broiler chicken litter fermentation with different fermentation lengths on organoleptic quality, and contents of cuprum (Cu), lead (Pb), worm eggs, fiber fractions including hemicellulose, cellulose, neutral detergent fiber (NDF), acid detergent fiber (ADF), lignin, and fermented litter fiber profile through analysis of scanning electron microscope-energy dispersive X-ray (SEM-EDX).

**Materials and Methods:**

This study used a complete randomized design of a unidirectional pattern with four treatments and four repeats with long fermentation treatments of 0, 3, 6, and 9 weeks.

**Result:**

The results showed a real influence (*p *≤ 0.05) of fermentation length on organoleptic quality, NDF, ADF, lignin, hemicellulose, and fiber profile with SEM-EDX observations, with no presence of worm eggs and heavy metal content is still at a safe level for feed materials.

**Conclusion:**

This study concluded that the processing of broiler chicken litter with 6 weeks of fermentation gave the best results on organoleptic observations, fiber profile, no presence of worm eggs, and heavy metal contamination that is safe for livestock.

## Introduction

Most of the ruminant farms in Indonesia are smallholding farms. The shortage of forage feed is a severe problem that farmers often face, both in quality and quantity. Indeed, forage supply in Indonesia depends on the season [1]. Feed forage in the rainy season is plentiful but rare during the dry season [2]. Farmers do not produce enough forage during the rainy season, so the absence of feed also happens in the dry season. The problem of feed availability may be solved by processing alternative feed materials with similar quality to forage and abundantly available during the dry season [3]. Beyond their availability, the problem related to alternative feed materials is the low quality and palatability of the feeds [4]. Among the alternative feed materials with good quality and abundant availability throughout the year is the litter of broiler chickens.

The litter of broiler chicken can be used as an alternative feed material because it contains crude fiber and high crude protein [5]. The litter of broiler chickens given directly to ruminant livestock is feared to interfere with livestock health. This is because litter is still feared to contain contamination of pathogenic microorganisms and heavy metals such as cuprum (Cu), lead (Pb), and mercury. These heavy metals can accumulate in the body and have toxic properties that can result in death in livestock [6]. Litter that is not handled correctly will cause wet and humid conditions, emit a foul smell, and result in parasites and fungi [7]. Processing of broiler chicken litter with fermentation can be used as a solution to reduce the content of crude fiber and contamination in broiler chicken litter [8].

The update of this study is the processing of broiler chicken litter through controlled fermentation technology that can be applied easily and cheaply among farmers. Fermentation litter processing is expected to reduce heavy metal contamination and prevent worm eggs and parasites that harm livestock. This study aimed to examine the effect of fermentation of broiler chicken litter with different fermentation lengths on organoleptic quality, and contents of Cu and Pb, the presence of worm eggs, fiber fractions that include hemicellulose, cellulose, neutral detergent fiber (NDF), acid detergent fiber (ADF), lignin, and fermented litter fiber profile through scanning electron microscope (SEM) analysis. The results of this study are expected to provide information about broiler chicken litter-based alternative feed that is safe, nutritious, and palatable for ruminant livestock.

## Materials and Methods

The materials used in this research were litter of broiler chickens, molasses, urea, salt, minerals, ExFeed starters, and distilled water. Methods used in the study included the preparation stage, the implementation of research, and the analysis stage. This research used a complete randomized design pattern in the direction of four treatments and four repeats. The treatments were as follows:

T0: broiler chicken litter fermentation of 0-week (0-day) acidification

T1: broiler chicken litter fermentation of 3-week (21-day) acidification

T2: broiler chicken litter fermentation of 6-week (42-day) acidification

T3: broiler chicken litter fermentation of 9-week (63-day) acidification

### Research procedure

#### Preparation stage

The research for this study began by collecting broiler chicken litter from 16 cages owned by Cemerlang Unggas Lestari Inc. A sampling of broiler litter was carried out by purposive random sampling method according to the capacity of each cage.

#### The research phase

Litter sampling is obtained by purposive random sampling based on cage capacity that can represent the cage’s percentage of area and capacity. Litter from each cage is weighed, weighing 2 kg, and mixed until homogeneous. The processing of litter as ruminant feed was using the fermentation process. In the fermentation method, litter that had been mixed from 16 cages was then divided into 16 parts weighing 1 kg each, then 60 ml molasses, 100 ml aqua dest/distilled water, 60 gm of salt, 60 gm starter ExFeed, and 60 gm of urea were added into each litter and mixed until homogeneous. According to the treatment, litter that had been homogeneous was inserted into the fermentor and fermented in a facultative anaerobe.

### Parameter testing stage

#### Organoleptic testing

Organoleptic testing using non-parametric analysis was carried out by a scoring method that observed and assessed contamination, smell, color, and texture based on a comparison scale [9]. The number of panelists in the study amounted to 20 people, with seven classes of comparison scale for organoleptic assessment.

#### Smell assessment

Score 1: the smell of ammonia is very pungent;

Score 2: the smell of ammonia stings;

Score 3: the smell of ammonia is slightly pungent;

Score 4: characteristic smell of ammonia;

Score 5: slight smell of ammonia;

Score 6: very little smell of ammonia

Score 7: odorless ammonia.

#### Texture assessment

Score 1: no blobs;

Score 2: very few blobs;

Score 3: slight blob;

Score 4: medium;

Score 5: more blobs;

Score 6: very many blobs;

Score 7: lump it all together.

#### Color assessment

**Figure 1.**
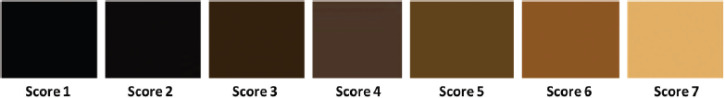
Color assessment parameters in organoleptic testing.

Score 1: deep black;

Score 2: black;

Score 3: dark brown blackish;

Score 4: dark brown;

Score 5: brown;

Score 6: light brown;

Score 7: yellow-brown (Fig. 1).

## Contamination assessment

Contamination covers all materials other than manure and husks, such as plastic, raffia rope, fur, insects, etc.

Score 1: there are six or more types of contamination,

Score 2: there are five types of contamination,

Score 3: there are four types of contamination,

Score 4: there are three types of contamination,

Score 5: there are two types of contamination,

Score 6: there is one type of contamination

Score 7: no contamination.

### Heavy metals testing

The heavy metals tested were for Cu and Pb. Testing of Cu content on the fermented chicken litter was carried out using the AAS method of flame. Cu-level measurement method using the AAS method is on, with a wavelength of 249.2 nm, acetylene/air flame type. Then, a standard solution is made and measured at wavelength so that its absorbance was visible. Furthermore, sample measurement was carried out in accordance with the procedure [10]. The process of testing the Pb content using the atomic absorption spectrophotometry method with different wavelengths for each type of metal being tested [4].

### Worm egg testing

Testing the content of worm eggs is carried out qualitatively and quantitatively. The qualitative testing process of worm eggs was using the floating method [11]. The principle of the floating method is to dissolve the sample that is suspected of containing worm eggs in a saturated sugar solution. In this method, the worm eggs will float if present. The process of testing the number of worm eggs was using the Whitlock method [12]. With this Whitlock method, the examination process was as follows: a sample weighing 3 gm was added into a saturated sugar solution of 60 ml and stirred until homogeneous. The soluble sample in the filter was then put in a plastic beaker. The filtrate was stirred, put in Whitlock chambers using pipettes until all the other Whitlock chambers are fully charged. Let the filtrate stay inside the Whitlock chambers for 5 min after it is ready to be examined under a microscope with a magnification of 4 × 10.

### Fiber fraction testing

Fiber fractions tested include neutral detergent fiber (NDF), ADF, and lignin levels. The ADF and NDF testing process was carried out based on the Van Soest method (1976) by boiling the sample in a solution of ADF and NDF then filtered over sintered glass with known weighing using hot water and alcohol. Strain them in the oven and weigh them. The lignin test was carried out using further testing of ADF and filtered using H_2_SO_4_. The hemicellulose content was obtained by reducing the NDF content with the ADF content in percent (%).

### Scanning electron microscope-energy dispersive X-ray (SEM-EDX) testing

SEM-EDX testing was conducted using SEM-EDX (SNE-4500) and Fourier Transform Infra Red (Perkin Elmer). The sample is pressed with a gold plating sputter and measured height by height. The specimen chamber in the mini SEM was opened, and the Z-height stur was adjusted to the height of the sample. Samples and stubs were placed on the stub sample seat. The specimen chamber was closed, the exchange button was pressed, and waited until the alarm sounded and the exchange light button stopped flashing. Live mode software Mini-SEM sets the focus of the tool according to the height of the sample. Then, adjust the position of the sample you wanted to see by using the X and Y axes. Samples were then tested at the laboratory to determine the composition of elements with SEM-EDX, in line with procedures [13].

### Data analysis

Data of heavy metals, worm eggs, and fiber fractions were analyzed using the analysis of variance test to test for data differences. Organoleptic data were analyzed using non-parametric analysis. If there is a significant effect, continue with the duncan multiple range test with a confidence level of 5%. [14]. SEM-EDX data were analyzed using descriptive qualitative methods.

## Results and Discussions

### Organoleptic quality, heavy metal content, and the presence of fermented chicken litter worm eggs at different ripening lengths

Average results of organoleptic and heavy metals tests on fermented chicken litter at different ripening lengths are shown in Table 1.

### Texture

Various analyses showed that fermentation in T2 treatment showed insignificant results with T3, but T1 was significant (*p* < 0.05) with T0. The average organoleptic value of fermented litter texture is 2.92. The value of 2.92 can be said that the fermentation of litter has a little texture of clots. Irfan et al. [15] state that fermentation in broiler chicken litter, in general, has a slight blob texture, juicy, and does not feel the initial texture. Factors that cause changes in texture in fermentation are temperature changes that result in changes in litter structure. In fermentation, there is a hot atmosphere that affects the structure of litter to become soft. Fermentation is a structural overhaul of the chemical, physical, and biological structure from complex to simple for an efficient digestibility for livestock feed [16,17].

**Table 1.** Average results of organoleptic and heavy metals tests on fermented chicken litter at different ripening lengths.

Parameters	Ripening lengths			
	T0	T1	T2	T3
Organoleptic quality				
Texture	1.99^c^ ± 0.07	2.99^b^ ± 0.22	3.29^ab^ ± 0.36	3.42^a^ ± 0.24
Color	2.92^c^ ± 0.35	2.97^c^ ± 0.40	5.25^a^ ± 0.46	4.45^b^ ± 0.50
Contamination	6,44^a^ ± 0.16	5.62^c^ ± 0.12	6.2^b^ ± 0.14	6.3^ab^ ± 0.04
Smell	2.81^ab^ ± 0.19	2.24^b^ ± 0.26	1.95^c^ ± 0.20	2.98^a^ ± 0.25
Heavy metal content				
Cu content (mg/kg)	42.56 ± 8.25	42.42 ± 5.68	49.89 ± 3.62	50.22 ± 10.24
Pb content (mg/kg)	5.17 ± 0.78	4.34 ± 0.44	4.46 ± 0.71	4.09 ± 0.49
The existence of worm eggs	Negative	Negative	Negative	Negative

The difference in superscript marks on the same line shows a noticeable difference (*p* < 0.05).

### Color

The analysis results show that fermentation in T0 treatment differed from the treatments of T1, T2, and T3 to the litter color of fermented chickens (*p* < 0.05). The average organoleptic value of fermented litter color is 3.9. A value of 3.9 indicates that fermented litter has a dark brown color. Irfan et al*.* [15] state that the litter of fermented husks generally has a blackish-brown color. The factor that causes discoloration is the change in temperature due to nitrogen (N) addition during fermentation. Aprintasari et al*.* [18] state that litter discoloration is caused by temperature changes due to the addition of N during fermentation causing color damage to litter. Temperature changes can occur during the fermentation process due to the activity of microorganisms in generating heat. Uhi [19] states that the increase in microorganisms is in line with the increase in fermentation temperature because microorganisms perform carbohydrate breakdown followed by energy release in heat, H_2_O, CO_2_ increasing fermentation temperature.

### Contamination

The results of various analyses showed that the contamination of fermented chicken litter in treatment T3 showed insignificant results with treatment T2 and treatment T0 but significant against treatment T1. The average organoleptic value of fermented litter contamination is 6.14. The value of 6.14 can be said that litter fermentation has one type of contamination. Caswell et al*.* [20] state that there are various contaminations of foreign bodies such as feathers and plastic on the litter of broiler chickens. Contamination in the fermented litter can be caused by incorrect handling. Illeghems et al*.* [21] add that the sorting process before fermentation needs to be done to separate foreign bodies so that the fermentation material is uniform. The results showed that the treatments of T2 and T3 have one contamination in the form of feathers. Chicken feathers on litter do not always impact the overall litter quality due to decomposition during fermentation. Rahayu et al*.* [22] state that chicken feathers contain fiber proteins (keratin), peptide bonds, and disulfide in keratin proteins that can be hydrolyzed by bacteria producing keratinase enzymes and reductase during the fermentation process.

### Smell

The results of various analyses showed that the smell of fermented chicken litter in the T0 treatment was no different from T1 and T3 treatments but had a real effect in T2. The average organoleptic value of fermented litter smell is 2.49. A value of 2.49 can be said that litter fermentation has a pungent ammonia smell. Caswell et al*.* [20] state that the fermentation process can reduce smell, weight, and volume. Smell changes can occur in the fermentation process into pungent ammonia due to a very strong degradation by fermented microorganisms. Wang et al*.* [23] state that fermentation results can produce a strong ammonia smell due to the degradation of carbohydrates, fats, amino acids, vitamins, minerals, pH, moisture, and smell ingredients by microorganisms. The smell of fermented broiler manure can be caused by microbial activity in the fermentation process. Jha and Berrocoso [24] states the smell of fermented products comes from the activity of microbial metabolism during the fermentation process.

### Cu content

The results of the analysis showed that the acidification time did not affect (*p *> 0.05) the copper (Cu) content in fermented chicken litter. The average content of copper metal (Cu) in the fermentation of broiler chicken litter is 42.42–50.22 mg/kg. Dierenfeld et al. [25] states that the need for Cu metal in feed is 50 mg/kg for cows and 15–20 mg/kg for sheep. Fermented chicken litter was still at a safe limit as feed can be given to cows. Erwiyansyah et al*.* [26] state the safe limit of Cu content in cow feed is a maximum of 100 mg/kg, and in sheep, feed is 25 mg/kg. Copper (Cu) plays a role in energy metabolism, nervous impulse transmission system, and immune system. Perera et al*.* [27] state that copper is included as a component of several enzymes that play a role in the formation of connective tissue, anti-oxidation, adrenaline hormone synthesis that produces energy, adrenaline hormone synthesis, red blood cell hemoglobin formation, maintaining central nerve function as well as helping the absorption of Fe elements. Cattle that lack Cu in the feed they consume will experience Cu mineral deficiency disease [28]. Olivia et al*.* [29] stated that environmental factors could also influence the high low content of Cu in broiler chicken litter. The factor is broiler feed contaminated with Cu content and then consumed by chickens, but the Cu content cannot be digested in the digestive tract to come out mixed with feces.

### Pb content

The analyses showed that the length of acidification did not affect (*p* > 0.05) the content of Pb in fermented chicken litter. Pb contamination in the chicken litter can come from chicken excreta or Pb contamination from husks due to metal contamination. Berata et al*.* [30] state that Pb metal contamination can be derived from nature and human activity. Poultry feed is generally derived from grain, Pb metal content in the feed material is not digested when consumed, so it is expelled with excreta. Fitriana et al*.* [31] stated that Pb metal in plants comes from pollutants in the atmosphere that can fall to the soil absorbed by roots and translocated to plants, while contaminants that fall on plants will be absorbed through stomata. The average metal content of Pb in chicken litter tested was 4.09–5.17 mg/kg. The results showed Pb metal content in the fermented chicken litter is classified as safe because it is below the limit. Salundik et al*.* [4] state that the standard content of Pb in feed is a maximum of 30 ppm. Contamination content that exceeds the limit can cause poisoning, health and performance disorders in livestock and humans who consume products from livestock containing Pb metal residues. Reckziegel et al. [32] states that the impact of Pb heavy metal contamination is accumulative in the body, causing a long-term impact on health, such as disruptions to the process of red blood cell formation. Contaminants can also influence Pb metal contamination in human activities by how the pollutants are discharged into the atmosphere [33].

### The existence of worm eggs

Based on the result of worm eggs content analysis in all treatments, no worm eggs were found. This indicates that fermented litter is safe for livestock to consume. Rinca et al. [34] states that cattle that have been infected with helminth eggs can be fatal because worm eggs will develop and absorb nutrients in cattle bodies. A closed house breeding system can minimize worm eggs in chicken litter. Bushra et al*.* [35] state that intensive maintenance and regular sanitation of cages can reduce the risk of helminth infection. It is further explained that changes in environmental conditions can affect parasitic infections such as helminth eggs [36]. Lalander et al*.* [37] stated that the growth of hookworms requires a temperature of about 35°C. Helminth egg infection is classified into three classes based on quantity, i.e., light class ranging from 0 to 500 eggs per gram (EPG), medium class which ranges from 501 to 1,000 EPG, and heavyweight with an amount exceeding 1,000 EPG [38].

### Profile of fermented chicken litter fiber at different ripening lengths

The flattening of fermented chicken litter fiber profiles at different ripening lengths is shown in Table 2.

### ADF levels

The analyses showed that the length of acidification had a natural effect (*p* < 0.05) on the ADF litter content of fermented chickens. The results of the T0 study differ markedly from T1, T2, and T3; T1 was no different from T3, while T2 was different from T1 and T3. ADF’s constituent components consist of easily digestible cellulose and hard-to-digest lignin. The highest ADF levels were in the T3 treatment. This happened because the NDF level in the T3 treatment was the lowest (34.32 ± 0.57), so it impacted the results of the T3 ADF level. Gomes et al*.* [39] state that the ADF is an insoluble part of the NDF in acid detergent. Low ADF levels in T0 indicated lower cellulose levels, so cellulose that could be used as an alternative energy source was also low. Putri et al*.* [40] state that cellulose is a constituent component of the ADF, so the lower the ADF content of an ingredient can occur due to lower cellulose levels.

**Table 2.** Average profile of fermented chicken litter fiber at different ripening lengths.

Parameters	Ripening lengths			
	T0	T1	T2	T3
ADF levels (%)	26.17^c^ ± 0.40	30.91^a^ ± 0.76	28.60^b^ ± 0.16	31.80^a^ ± 0.93
NDF levels (%)	40.11^a^ ± 0.54	37.91^b^ ± 0.44	36.60^c^ ± 0.35	34.32^d^ ± 0.57
Lignin levels (%)	6.91^b^ ± 0.37	6.53^b^ ± 0.31	6.63^b^ ± 0.46	7.66^a^ ± 0.36
Hemicellulose levels (%)	13.94^a^ ± 0.34	7.01^b^ ± 0.55	8.00^c^ ± 0.43	2.52^d^ ± 0.74

Different superscripts on the same line show a noticeable difference (*p* < 0.05).

The fermented chicken litter ADF value in all treatments was still in the safe range to be given to livestock with ADF levels (26.17%–31.80%). Tavirimirwa et al*.* [41], as cited in Tavirimirwa et al*.* [41], stated that the percentage of ADF in feed materials that could be given to livestock is 25%–45%. Further explained by Lesmana et al*.* [42], the content of ADF that is good for livestock is below 30% because the low levels of crude fiber make high digestibility. In comparison, ADF values that are more than 35% will decrease the digestibility of feed.

### NDF levels

The results of the variance test showed that the curing time had a significant effect (*p* < 0.05) on the NDF levels of fermented chicken litter. The ripening was done to reduce NDF levels. NDF levels at T0 were significantly different from NDF levels at T1, T2, and T3; NDF levels at T1 were substantially different from NDF levels at T2 and T3; NDF levels at T2 were significantly different from NDF levels at T3. The decrease in NDF levels occurred because there were lactic acid microorganisms that digested the complex components such as lignin, cellulose, and hemicellulose from chicken litter into simpler components during the fermentation process. Usman et al*.* [41] state that microbes that digest the cell walls of the material during fermentation can reduce NDF levels. Examples of NDF complex components are lignin, cellulose, and hemicellulose [43]. NDF constituent components that are too high will cause low material dilution. NDF comprises silica, cellulose, pectin, hemicellulose, cutin, and lignin, which are difficult to digest.

The real difference in NDF levels between each treatment was thought to be due to the long period between one and the other, i.e. 3 weeks so that the time for microorganisms to break down lignin and hemicellulose was longer. NDF levels that were still high at T0 were due to the absence of microorganisms to break down cellulose, lignin, and hemicellulose. Setiawan and Mansyur [44] state that the decreased NDF levels could be due to the separation of lignin and hemicellulose carried out by microbes. The duration of curing affected NDF levels at T2 and T3 because the time needed to digest hemicellulose and cellulose completely was sufficient. The longer the curing time, the lower the NDF level of fermented chicken litter. Low lignin levels in chicken litter also affect the level of NDF levels. This is related to the ability of microorganisms during fermentation because the higher the lignin level, the lower the ability of the microorganisms to break down the hemicellulose and cellulose components in the material, thus making the NDF levels higher. Sudirman et al*.* [45] state that increasing lignin levels can decrease hemicellulose levels so that microcellulose cannot decompose both hemicellulose and cellulose. High levels of NDF indicate that the fiber components that make up the ingredients are also high, thereby reducing the digestibility of the material when consumed by livestock. Hambakodu et al*.* [46] state that the quality of the feed can be measured by the digestibility level since the high fiber fraction component will reduce the digestibility of the feed in the rumen.

### Lignin levels

The results of the variance test showed that the curing time had a significant effect (*p* < 0.05) on the lignin litter content of fermented chicken. The lignin levels at T0 were not significantly different from the lignin levels at T1, T2, and T3; lignin levels at T1 were the same as T2, and both were substantially different from lignin levels T3. The analysis results show that there is no effect of long fermentation with control treatment or without fermentation, presumably because lignin could not be digested by microorganisms so that the lignin levels in all treatments were relatively the same. Fitiani et al*.* [47] state that lignin is a component that binds strongly to hemicellulose and cellulose and cannot be digested by microorganisms. The fermentation process of chicken litter using lactic acid bacteria under facultative anaerobic conditions also affected the different lignin levels between T0, T1, T2, and T3 treatments. The lactic acid bacteria present during the fermentation process was thought to be unable to degrade lignin. Shrivastava et al. [48] state that lignin is a part that cannot be digested, which results in low digestibility of the material, where the lignin degradation process requires a different process.

The recommended treatment was at T2, i.e. curing the fermented chicken litter for 6 weeks because it provided the same lignin content as T1 and T0 treatments. The curing treatment that was not recommended was T3, i.e. curing the fermented chicken litter for 9 weeks because the lignin content reached 7.66% compared to other treatments. Tatra et al*.* [49] stated that the limit of 7% lignin content in feed ingredients can still be tolerated by livestock when the feed material is consumed. The higher lignin indicates that the digestibility of the material will decrease because lignin is a component of fiber that is difficult to degrade by microorganisms. Sitorus [50] stated that high lignin levels result in low crude fiber digestibility.

### Hemicellulose levels

The results of the variance test showed that the curing time had a significant effect (*p* < 0.05) on the hemicellulose levels of fermented chicken litter. The ripening was done to reduce hemicellulose levels. Hemicellulose levels at T0 were significantly different from hemicellulose levels at T1, T2, and T3; however, the hemicellulose levels at T1 and T2 were not significantly different; and hemicellulose levels of T1 and T2 were significantly different from T3. The decrease in hemicellulose content occurred due to the fiber fraction factor. Hemicellulose is a component of fiber that is easily digested by microorganisms into glucose products. The longer curing time in the fermented chicken litter will be followed by a lower value of the hemicellulose content produced. Definiati et al*.* [51] state that microorganisms can digest and break down hemicellulose into simple molecules, resulting in decreased hemicellulose content. Hemicellulose in the fermented chicken litter is lower, presumably due to the shorter length of the polymer and the length of fermentation coupled with the alkaline soluble hemicellulose. Nurkhasanah et al. [52] state that hemicellulose can dissolve in alkaline at low concentrations and the higher the solubility of hemicellulose is due to the more branches.

The levels of hemicellulose in the fermented chicken litter were influenced by the levels of ADF and NDF, because the levels of hemicellulose were obtained from the reduction of the levels of NDF to levels of ADF. Nurjanah et al*.* [53] state that hemicellulose is a heteropolymer polysaccharide and a component of the cell wall obtained from the difference between NDF and ADF. The recommended treatment is at T3, i.e. curing the fermented chicken litter for 9 weeks, because it provided the best hemicellulose content results compared to other treatments. The low hemicellulose content indicates that the cellulose-binding component in the material is also low, so the digestibility of the material will be high. Siregar et al*.* [54] state that hemicellulose naturally binds cellulose, so that low hemicellulose will make cellulose an alternative source of energy because it can be converted into glucose.

### Fiber profile through SEM-EDX observation

The observation results of the composition of fermented chicken litter elements at different ripening lengths using SEM-EDX are shown in Table 3 and Figure 1.

From Table 3, it is known that the element composition of carbon (C) in the litter is 40.81%–45.58%. The composition of element C in the non-fermentation treatment (T0) and the fermentation treatment (T1, T2, and T3) is almost in the same range because the litter material contains cellulose as a C source. Purbowatiningrum et al. [55] states that the generally used C source in fermentation is cellulose because it is easily obtained and undergoes hydrolysis through enzymatic and chemical processes. The N element in litter without fermentation was 10.19% by weight, while there was no N element in the fermentation treatment. The N element counted in the non-fermentation treatment came from urea. Meanwhile, after fermentation, the N element was not found because microorganisms used it as a substrate during the fermentation process. Sundari et al*.* [56] state that they need a source of C and N for the living activities of microorganisms.

**Table 3.** Composition of fermented chicken litter elements at different ripening lengths.

Elemental composition	Ripening lengths			
	T0	T1	T2	T3
	%			
C	42.53	40.81	41.4	45.58
N	10.19	–	–	–
Na_2_O	1.04	0.83	0.92	1.16
MgO	0.36	–	–	0.48
Al_2_O_3_	0.52	–	–	0.47
cSiO_2_	38.64	56.45	54.63	48.77
P_2_O_5_	0.78	–	–	0.81
SO_3_	0.76	–	–	–
Cl	1.49	0.83	1.32	0.89
K_2_O	1.33	0.88	1.05	1.00
Calcium oxide (CaO)	1.21	0.21	–	0.85
CuO	1.15	–	–	–
MgO	42.53	–	–	–
Al_2_O_3_	10.19	–	–	–
cZnO	–	–	0.95	–

Sodium oxide natrium oxide (Na_2_O) elemental composition in a litter ranges from 0.83%–1.16% by weight. The fermentation treatment carried out did not have a significant effect on the presence of Na_2_O. Na_2_O cannot be decomposed by fermentation microorganisms so that the amount is relatively the same in all treatments. Wahyuningsih et al. [57] state that Na_2_O is one of the constituent components of zeolite or hydrated aluminosilicate compounds from alkaline earth, and alkaline metals when heated release water content. Elements of magnesium oxide (MgO) and alumina (Al_2_O_3_) were not found in T1 and T2 treatments but were found in T0 and T3 treatments. The MgO levels at T0 and T3 were 0.36% and 0.48%, respectively, while the Al_2_O_3_ levels were 0.52% and 0.47%. The T1 and T2 fermentation treatments did not contain MgO and Al_2_O_3_ elements because the treatment produced bioethanol (liquid from the fermentation process), and the resulting bioethanol reacted with these two elements. Ayuti et al. [58] state that bioethanol can react with aluminum-magnesium metal, and the higher the fermentation temperature, the lower the bioethanol produced. This is also the reason that in the T3 treatment, there are elements of MgO and Al_2_O_3_ because it is suspected that the temperature is higher so that the production of bioethanol is low.

**Figure 2.**
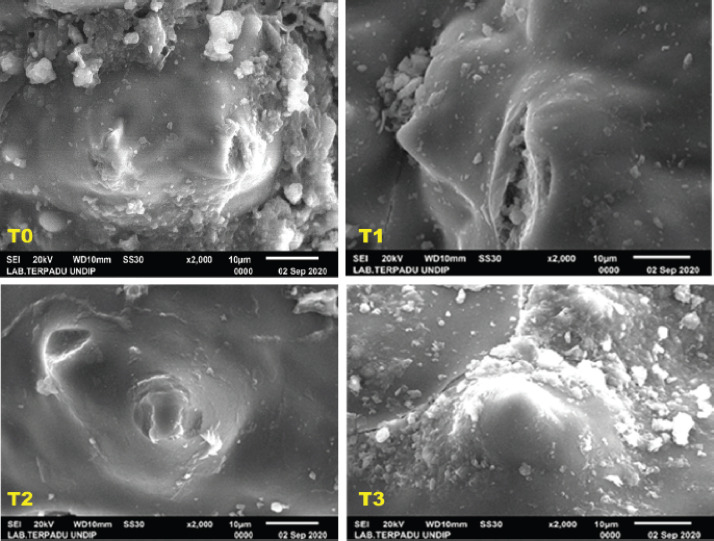
Composition of fermented chicken litter elements at different ripening lengths using SEM-EDX SEM. Zoom image test 2,000×

The composition of silica dioxide (SiO_2_) in subsequent studies from lowest to highest was T0 (38.64%), T3 (48.77%), T2 (54.63%), and T1 (56.45%). SiO_2_ litter comes from rice husk. The decomposed husk during fermentation resulted in SiO_2_ production at T1 and T2 greater than the treatment. SiO_2_ in litter has a positive impact as an additive to prevent clumping of litter products. Putra et al*.* [59] state that silica or SiO_2_ is a source of anti-baking ingredients in the food that can be produced from rice husks because of its more reactive, refined form, low cost, and renewable properties. Phosphorus pentaoxide (P_2_O_5_) is found in T0 (0.78%) and T3 (0.81%), while the sulfite (SO_3_) element is only found in T0 (0.76%) treatment. P_2_O_5_ and SO_3_ were not found in the T1 and T2 fermentation treatments because bacteria had synthesized these elements during fermentation. Meanwhile, P_2_O_5_ was found in the T3 fermentation treatment, presumably due to increased phosphorus-decomposing bacteria. Therefore, this Pbs to an increase in P_2_O_5_ which does not work properly due to the long process of fermentation. Cesaria et al*.* [60] stated that an increase in fermentation microorganisms could increase phosphorus levels from P_2_O_5_.

The elemental composition of chloride (Cl) and potassium oxide (K_2_O) from lowest to highest is T1 (0.83%; 0.88%), T3 (0.89%; 1.00%), T2 (1.32%; 1.05%), and T0 (1.49%; 1.33%). Fermentation treatments (T1, T2, and T3), when compared to non-fermenting treatments (T0) experienced a relative decrease in Cl and K2O elements, this is presumably because these elements during fermentation have been used as substrate components by fermenting microorganisms. Ratrinia et al*.* [61] state that K_2_O levels can increase during fermentation due to cell division by microorganisms. The highest elemental composition of calcium oxide (CaO) was found in the T0 treatment (1.21). In contrast, in the fermentation treatment, the lower CaO elements were T1 (0.21), T3 (0.85), and no CaO element in the T2 treatment. The composition of CaO and Cl decreased in the fermentation treatment because they were used as essential food elements for the metabolism of microorganisms. Pasaribu [62] states that to grow and develop, microorganisms require macronutrients and micronutrients in the form of trace elements such as calcium chloride and Cobalt chloride hexahydrate.

The elemental compositions of copper (II) oxide (CuO), MgO, and Al_2_O_3_ were only found in the treatment without fermentation (T0) with an amount of 1.15%, 42.53%, and 10.19%, respectively. Meanwhile, these three elements were not found in all fermentation treatments (T1, T2, and T3). This is thought to occur because these elements have been degraded by fermenting microorganisms as a medium for survival. Pasaribu [62] states that magnesium in fermentation media can be used as a macronutrient by fermentation microorganisms.

Zinc oxide (ZnO) element was only found in the T2 treatment at 0.95%. Wientarsih et al*.* [63] state that ZnO is a form of zinc mineral that is not toxic even though it is used at relatively high doses. Based on the SEM-EDX analysis, the 6-week fermentation time (T2) treatment showed the best results because the composition of the elements that fermenting microorganisms had decomposed was more optimal. When given to livestock as feed, it would minimize potential disturbances. T2 treatment also contains ZnO, which can be an additional source of minerals in the feed.

## Conclusion

Fermented broiler chicken litter processing can provide the best organoleptic results, lower fiber fractions, and a safe composition of elements and contamination for ruminant livestock with the best fermentation duration at 6 weeks.

## List of Abbreviations

ADF: Acid Detergent Fiber; Al_2_O_3_: Alumina; C: Carbon; CaO: Calcium Oxide; xCu: Cuprum; CuO: Copper (II) Oxide; EPG: Eggs Per Gram; K_2_O: Kalium Dioxide; MgO: Magnesium Oxide; N: Nitrogen; Na_2_O: Natrium Oxide; NDF: Neutral Detergent Fiber; P_2_O_5_: Phosphorus Pentaoxide; Pb: Lead; SEM: Scanning Electron Microscope; SEM-EDX: Scanning Electron Microscope-Energy Dispersive X-ray; SiO_2_: Silica Dioxide; SO_3_: Sulfite; ZnO: Zinc oxide.
